# Liposomes and transferosomes in the delivery of papain for the treatment of keloids and hypertrophic scars

**DOI:** 10.1371/journal.pone.0290224

**Published:** 2023-12-15

**Authors:** Rachana Sapkota, Daniel J. Munt, Anthony E. Kincaid, Alekha K. Dash

**Affiliations:** Department of Pharmacy Sciences, School of Pharmacy and Health Profession, Creighton University, Omaha, Nebraska, United States of America; Siksha O Anusandhan University School of Pharmaceutical Sciences, INDIA

## Abstract

Hypertrophic scars and keloids are characterized by an excessive collagen deposition. The available treatment options are invasive and can result in recurrence of scar formation. Using liposomes and transferosomes for the topical delivery of papain, a proteolytic enzyme, can be effective treatment. The objective of the study is to formulate papain-loaded liposomes and transferosomes, characterize the formulations, and study *in vitro* permeation using shed snake skin and Sprague-Dawley rat skin as models for stratum corneum and full thickness skin. Papain-loaded liposomes and transferosomes were formulated using the thin-film hydration method for the delivery of papain across the stratum corneum barrier. An *in vitro* permeation study carried out using shed-snake skin and Sprague-Dawley rat skin models showed that transferosomes were able to deliver papain across the stratum corneum barrier, while papain solution and papain liposomes were not able to cross the barrier. However, transferosomes were not able to deliver papain across the full thickness rat skin model suggesting the deposition of papain loaded transferosomes in the epidermal or dermal layer of skin. In addition, an ex-vivo model was used to analyze the effect of papain exposure on the morphology of the epidermis taken from rat skin exposed to papain solution, papain in transferosomes and papain in liposomes. Papain in solution resulted in a noticeable degradation of the epidermis, but when embedded in either transferosomes or liposomes there was no noticeable change when compared to control animals. The cytotoxicity study performed using HeLa cells showed that the cells were viable at papain concentrations lower than 0.01 mg/ml.

## 1. Introduction

Skin is the largest organ of the human body and it serves multiple purposes. It acts as a physical barrier offering protection from light and foreign substances. It also regulates body temperature and is involved in the synthesizes vitamin D [1–3]. Skin is comprised of epidermis, and dermis. The stratum corneum, a part of the epidermis, is the most superficial layer of skin. It is thought to act as a barrier for topical and transdermal delivery of drugs [4]. The dermis is a layer underneath the epidermis and is composed primarily of extracellular matrix of collagen and elastin fibers with a relatively modest number of cells. The major component of the extracellular matrix is type I collagen, which makes up 70% of the total weight of dermis [5, 6].

The excessive collagen deposition in the dermal layer of the skin during the healing process after an injury can give rise to fibro-proliferative disorders such as hypertrophic scars and keloids. These conditions arise after trauma to the dermis inflicted by burns, surgery, tattoo, acne, vaccinations, and other skin damaging penetrations [7, 8]. Keloids are more aggressive compared to hypertrophic scars and grow outside the boundary of the original wound [9]. Hypertrophic scars and keloids are observed more often in African, Asian, Mediterranean, and Hispanic population compared to Caucasians. This may be due to the involvement of melanocyte stimulating hormone [10]. Carswell and Borger studied the family history of patients with keloids and hypertrophic scars and concluded that genetic factors may play a role in the development of hypertrophic scars and keloids [11]. Hypertrophic scars and keloids are cosmetically disfiguring to patients, causing social stigma and lack of confidence. There are multiple treatment options available for the treatment of hypertrophic scars and keloids, but many of the available options are painful, ineffective and contribute to the reccurrence of the scars [12–14].

Since these scars arise due to the uncontrolled growth of collagen, proteolytic enzymes such as papain may be effective in the treatment of hypertrophic scars and keloids. Papain is a cysteine protease enzyme obtained from the latex of raw papaya (*Carica papaya* L.) fruit [15]. It is reported to have proteolytic, antimicrobial, anti-inflammatory, antibacterial, and anticancer properties [16–18]. Papain has 212 amino acids with three disulfide bridges and the molecular weight is 23,406 Da [19]. Three amino acids, Cys25, His158 and His159, present in papain are responsible for proteolytic activity. The cleavage of the amide bond in protein and peptide is reported to be the specificity of papain [20]. Further details on the mechanism of action for the proteolytic effect of papain are discussed in the literature [21].

Papain is a large molecule that cannot cross the stratum corneum to reach the dermal layer of the skin [22]. It needs assistance in transportation. Liposomes and transferosomes are candidates could be utilized in the delivery of papain by overcoming the stratum corneum barrier. Liposomes are vesicles made up of phospholipid bilayers with hydrophilic core. They are capable of encapsulating both hydrophilic and hydrophobic entities [23–25]. Liposomes contain cholesterol embedded in the phospholipid bilayer, causing the bilayer to stabilize and gain stiffness. This restricts the passage of liposomes to the deeper layer of the skin. Transferosomes are flexible vesicles with edge activators embedded in the phospholipid bilayer. Transferosomes were initially used as delivery tools for topical and transdermal analgesics. They are similar to liposomes in their structural aspects but are unique in their functional aspect [26–29]. The hypothesis tested by this study is that both liposomes and transferosomes effectively encapsulate and deliver papain across the stratum corneum barrier when applied topically.

## 2. Materials

Papain, for biochemistry, was purchased from ACROS Organics (New Jersey, USA). Acetonitrile and formic acid were purchased from Fisher Scientific (Fair Lawn, USA). The Synergi 4u Polar-RP 80A column was obtained from Phenomenex (Torrance, CA, USA). Deionized (DI) water was used in the preparation of the samples and standard. Soy lecithin was purchased from Alfa Aesar (India). Diethyl ether was purchased from Sigma Aldrich (Saint Louis, MO). Tween 80, cholesterol, and D-trehalose dihydrate was purchased from (Fisher Scientific Fair Lawn, NJ).

The shed snake skin was purchased from Omaha’s Henry Doorly Zoo. Dr. Justin Tolman’s lab provided the Sprague Dawley rat skin at Creighton University. HeLa cells were purchased from the American Type Culture Collection (ATCC) (Manassas, VA). Blood used for the hemolysis study was single donor human whole blood obtained from a Caucasian male (Age: 39 years) and purchased from InnovativeTM Research (Novi, MI).

Eagle’s Minimum Essential Medium (MEM), penicillin-streptomycin, L-glutamine, and sodium pyruvate were purchased from CellGro® (Manassas, USA). MTT was purchased from Acros Organics (New Jersey, USA), and, N, N, dimethyl formamide (DMF), Gibco® 0.25%, trypsin-EDTA, and sodium dodecyl sulfate (SDS) were purchased from Thermo Fisher Scientific (Fair Lawn, USA). Fetal bovine serum (FBS) was purchased from Atlanta Biologicals (Flowery Branch, GA). Blue dextran and Dubelco’s phosphate buffered saline were obtained from Sigma-Aldrich (Saint Louis, MO). Lucifer yellow CH, lithium salt, was purchased from Molecular Probes (Eugene, OR). NairTM was purchased from a local drug store.

## 3. Methods

### 3.1. Analytical method in HPLC

An analytical method was developed employing reversed phase HPLC on a 150 x 4.6 mm column maintaining the column temperature at 40 C. The mobile phase consisted of a mixture of 0.1% v/v formic acid in acetonitrile (25%) and 0.1% (v/v) formic acid in water (75%). The apparent pH of the mobile phase was recorded as 3.04 using the Accumet basic pH meter (Fisher Scientific). The flow rate was maintained at 1.0 ml / min. The detector was a photodiode array detector (PDA), and the detection wavelength was 280 nm. The maximum absorbance of the protein was observed at 280 nm due to amino acids such as tyrosine and tryptophan, which contain aromatic rings with Π (pie) electrons. Increasing resonance inside the aromatic ring causes the lambda maximum for most proteins, including papain, to be at 280 nm. Chromatographic conditions are summarized in Table 1. The developed method was validated for specificity, linearity, precision, and accuracy to comply with the United States Pharmacopoeia (USP).

**Table 1:** Particle size of blank papain and papain loaded vesicles (Liposomes and transferosomes) before lyophilization (pre-lyo) and after lyophilization (post-lyo) with and without trehalose.

Samples	Particle size (nm) (Pre-lyo)	Particle size (nm) (Post-lyo)	Particle size (nm) (Post-lyo with trehalose)
Blank liposomes	140.09 ± 0.09	578.90 ± 9.30	159.40 ± 5.40
Papain liposomes	150.00 ± 2.30	475.60 ± 9.60	160.80 ± 6.70
Blank transferosomes	156.00 ± 3.60	237.60 ± 3.50	140.20 ± 2.10
Papain transferosomes	158.00 ± 3.50	238.00 ± 1.10	147.70 ± 2.50

The zeta potential value of liposomes, transferosomes, both blank and loaded with papain is anionic (-50.09±3.66 for papain liposomes and -43.51±1.73 for papain transferosomes).

### 3.2. Preparation of liposomes and transferosomes

The thin-film hydration method was chosen because of the ease of preparing liposomes and transferosomes on a laboratory scale. Papain-loaded liposomes and transferosomes were prepared using the thin-film hydration method. For liposomes, 200 mg soy lecithin and 50 mg cholesterol were taken in a 100 ml round bottom flask and 20 ml of ether was added to dissolve lipids. Similarly, for transferosomes, 200 mg of soy lecithin and 50 mg of tween-80 were added with 20 ml of ether. The ratio of soy lecithin and cholesterol/tween 80 was optimized for particle size. Liposomes and transferosomes were prepared using a thin-film hydration method and particle size was analyzed using dynamic light scattering (DLS). Cholesterol is added to soy lecithin because the incorporation of cholesterol into the bilayers of phospholipids makes the bilayer rigid and ensures the structural integrity of the vesicles [30]. The highly flexible and non-bulky hydrocarbon chains present in tween 80 are attributed to the production of higher flexibility, high drug encapsulation, and smaller size particles compared to spans and bile salts [31, 32]. Therefore, tween 80 was chosen as an edge activator for this study.

The round bottom flask was then mounted on a rotary evaporator. A thin film of lipids was obtained after ether was removed under reduced pressure. The film was stored under vacuum overnight and then hydrated with 100 mM phosphate buffer at pH 7 containing papain to obtain a suspension. This mixture was shaken for a few minutes and then ultrasonicated for 15 min.

Furthermore, the suspension was extruded using an Avanti polar lipid mini extruder. 11 passes of the suspension were performed across a 0.2 μm polycarbonate membrane. The formulation was further freeze-dried to obtain it in powder form. For freeze drying, the shelf temperature was maintained at -50°C for 240 min, and the final freeze was carried out at -60°C for 20 min. After freezing, -20 ˚C was maintained for 265 min, -10 ˚C for 270 min, 0 ˚C for 270 min, 10 ˚C for 270 min, 20 ˚C for 390 min. The shelf temperature was maintained at 20˚C for 360 min for secondary drying. The vacuum was maintained at approximately 662,000 millitorrs.

### 3.3. Determination of the particle size and zeta potential

To observe the particle size and zeta potential of the samples, they were diluted 10 times with 0.2 μm filtered DI water. The particle size and zeta potential of liposomes and transferosomes were determined using the Brookhaven Zetameter (Zetaplus, Brookhaven Instruments Corporation, Holtsville, NY) which is based on the principle of dynamic light scattering. Additionally, the particle size stability of liposomes and transferosomes was observed over 72 hours to evaluate the particle size stability of the nanoparticles.

### 3.4. Evaluation of drug loading and encapsulation efficiency of liposomes and transferosomes

The liposomes and transferosomes in suspension were ultracentrifuged at a force of 328,000 G for 20 minutes using the SORVALL Discovery 90SE ultracentrifuge. The supernatant was collected, and the pellet at the bottom of the ultracentrifuged tube was washed with DI water three times and three washings were collected. The analytical method in HPLC was used to quantify the papain present in the washings. The amount of papain in the formulation was calculated by subtracting the amount quantified in the supernatant layer from the total amount added.

### 3.5. Transmission electron microscopy (TEM) of liposomes and transferosomes

Particles (∼20 μL) of ∼0.1 mg/ml concentration 0.1 mg / ml were placed on carbon-formvar-coated copper grids. Copper grids were activated immediately prior to use by UV irradiation for 30 minutes. The copper grid loaded with particles was kept at room temperature for 30 minutes. An adsorbent tissue was used to gently blot the grids. Negative staining of the sample was performed by adding 20 μl Osmium tetroxide (0.25% w/v) on top of the grids and drying at ambient temperature under vacuum.

### 3.6. Enzymatic activity of papain

To determine the enzymatic activity of papain, protease assay was performed. Casein was used as a substrate in this assay. Preparation of the Casein solution (10mg/ml) was performed using an EDTA (ethylene diamine tetraacetic acid) L-cysteine buffer. L-cysteine EDTA buffer was prepared by mixing 7.0 g of EDTA, 3.55 g of dibasic sodium phosphate and 3.05 g of L-cysteine monohydrate in 500 ml of DI water. Then the casein solution was incubated at 40˚C for 30 minutes in microcentrifuge tubes. The samples (papain, liposomes and transferosomes containing papain) were added to the tubes and further incubated at 40˚C for an hour. Furthermore, trichloroacetic acid (30% w/v) was added to stop papain and casein reaction. Casein, sample (papain), and trichloroacetic acid were added in a ratio of 5:2:3 (v/v). For blanks, samples (papain) were added after the addition of trichloroacetic acid. The microcentrifuge tubes were chilled at 4 ˚C for 30 minutes and centrifuged for 10 minutes at 17,000 G force using a microcentrifuge (AccuSpin™ Micro 17, Thermo Fischer Scientific, USA). The supernatant collected after the spin was collected and pipetted into a 96-well glass plate. The pellets formed at the bottom of the tubes were not disturbed during the process. The samples were run in triplicates along with the appropriate blanks. The 96-well glass plate was analyzed under ultraviolet light at 280 nm. Control for the lipid in the papain containing vesicles (liposomes and transferosomes) was carried out by running blank liposomes and transferosomes. The absorbance from the blank vesicles was subtracted from the absorbance of papain-loaded liposomes and transferosomes. The enzymatic activity produced by the papain solution was considered to be 100%, and the percentage of enzymatic activity produced by the papain-loaded liposomes and transferosomes is compared to the papain solution.

### 3.7. In vitro permeability studies of papain-loaded liposomes and transferosomes on shed snake skin

The capacity of a drug to cross the physiological barrier is called permeability. Shed snake skin was used as a model for stratum corneum to study the *in vitro* permeability of papain from aqueous solution, liposomes, and transferosomes. Shed snake skin was prepped by washing it in tap water and then by DI water. It was cut lengthwise on the belly portion to split it open. The skin was then suspended on the water in a way that the inner side of it was towards the water. A sheet of paper was slid underneath the skin as it suspended in water and the skin was mounted into the paper. This was done to make the marking, cutting, handling, and storage of the skin easier. The skin was dried along with the paper and then appropriate marks and cuts were made to fit the Franz cell. The skin sections were stored in an airtight ziplock bag at room temperature. The skin was rehydrated by soaking them in overnight in DPBS at 4 ˚C prior to use.

In a Franz diffusion cell, a shed snake skin was placed between the donor and receptor compartment. The sample was exposed to 0.67 cm^2^ of skin. The receptor compartment was filled with 5.2 ml of 0.1 M DPBS at pH 7.4 as a medium. The Franz cell temperature was maintained at 32°C to resemble the skin temperature. A magnetic stirrer was placed in the receptor compartment to stir the media. The skin was placed between the receptor and the donor compartment. It was allowed to equilibrate at room temperature for 20 minutes. The 200 μL sample was then added to the donor compartment and 200 l samples of 200 μL were collected at 15 min, 30 min, 60 min, 90 min, 120 min, 180 min, 240 min, 300 min. After each sampling, 200 μL of the volume was replaced with DPBS. The samples were filtered through a 0.2 μm syringe filter and further analyzed using HPLC.

As the skin of the shed snake is a thin and fragile membrane, it can lose integrity during handling and experiment, so blue dextran was used to check for it. The blue dextran solution (1 mg/ml) was prepared in DPBS and added to the donor compartment at the end of the study. The sample was collected after an hour and analyzed using a UV-visible spectrometer at 620 nm.

### 3.8. In vitro permeability studies of papain-loaded liposomes and transferosomes on Sprague-Dawley rat skin

In vitro permeability of papain from papain in aqueous solution, liposomes, and transferosomes was performed using full thickness dorsal skin of a female Sprague-Dawley rat. After inhalation of a placebo dose of lactose, rats were sacrificed using isoflurane. The gender and part of the body from which the skin was extracted were kept uniform due to the variation in the thickness of the skin between genders and different parts of the body. The skin, along with the smooth muscle layer and connective tissue, was extracted from the animal. The smooth muscle layer underneath the skin was scraped with a knife.

The hair was partially removed using an electric hair clipper followed by applying NairTM (sodium hydroxide-containing hair removal cream) for 3 minutes. The duration of application for hair removal cream was optimized based on the integrity of the stratum corneum, which was visually observed. The skin was rinsed with running tap water to remove residual cream. It was further washed with DI water and makings were made to cut skin in appropriate sections for the Franz cell. A metal cylinder with approximately the same diameter as the Franz cell was used as a measure, and scissors were used to cut sections. The skin was used the same day and the following day to maintain the integrity of the physiological barrier. Until used, skin was stored in 0.1 M DPBS solution at 4 ˚C.

Skin was sandwiched between the donor and receptor compartments in the Franz diffusion cell. The area of the skin exposed to the sample and receptor medium was 0.67 cm2. The medium used for this study was 0.1 M DPBS at pH 7.4. An aqueous papain solution and formulation were prepared in DPBS. Franz cells were equipped with an acrylic jacket, which is circulated with water. The system temperature was maintained at 32 C, which resembles the temperature of the skin. The volume of the Franz cell receptor compartment was 5.2 ml. The medium was added to the receptor compartment and the shed snake skin was sandwiched between the compartments with the help of gaskets. 200 μL sample was placed in the donor compartment. 200 μL were collected from the sampling arm at 15 min, 30 min, 60 min, 90 min, 120 min, 180 min, 240 min, 300 min and the volume of the withdrawn sample was replaced with 200 μL of medium. The collected samples were filtered using a 0.2 μm syringe filter and further analyzed using the HPLC method.

A lucifer yellow solution (100 μg/mL) prepared in DPBS was added to the donor compartment at the beginning of the study. Although skin was used within 48 hours after extraction from the animal, it could undergo necrosis and lose its physiological integrity. Therefore, lucifer yellow, a small hydrophilic molecule, was added as a tracer to establish an intact physiological barrier. It is a highly sensitive molecule. The receptor medium was collected as a sample and was analyzed for fluorescence with the excitation maximum at 428 nm and emission maximum at 534 nm.

### 3.9. 3-(4, 5-dimethylthiazolyl-2)-2, 5-diphenyltetrazolium bromide (MTT) cytotoxicity assay

The MTT cytotoxicity assay was performed for an aqueous solution of papain and papain-loaded vesicles. Blank particles were matched to the lipid concentration in papain-loaded vesicles. The effective dose of papain for the treatment of hypertrophic scars is not clearly illustrated in the literature. Therefore, a wide range of concentrations was studied to analyze a safe concentration. The samples were prepared using freeze-dried powder. For this study, five papain concentrations and papain-loaded vesicles were prepared in the range of 0.001 mg/ml– 10 mg/ml.

A nonkeratinized epithelial cell line, HeLa, was used for this experiment. Cells were grown using MEM supplemented with 10% FBS, 1 mM sodium pyruvate, 0.1 mM non-essential amino acids, 2 mM L-glutamine, 100 μg/ml. A 75 cm^2^ flask was used as the main plate to grow the cells.

After incubation, treatment solutions were removed, and cells were supplemented with MEM. The MTT solution was prepared by dissolving the MTT reagent in serum-free MEM at a concentration of 1 mg / ml. After incubation of 24, 48, and 72 hours, the medium was removed from the cells and then 100 μL of MTT solution was added to the cells and incubated for 4 hours. 100 μL of a 1:1 solution of 20% (w/v) SDS and DMF was added. Plates were shaken on a shaker (Big Bill, thermolyne) for one hour at 37°C. The absorbance was measured by spectroscopic analysis using a microplate reader (Thermo Scientific) at 540 nm. All studies were performed in triplicate.

### 3.10. Ex-vivo histologic analysis of the effects of papain on skin morphology

Full-thickness skin from female Sprague-Dawley rats was collected from 21 animals following terminal euthanasia using isoflurane. Round tissue sections with a diameter of approximately 3.2 cm were marked using a metal cylinder and cut using scissors. DPBS was added in a 6 well cell culture plate and the skin patch was suspended on top of it. 1 ml of sample (liposome, transferosome, papain, and DPBS) at 1mg/ml concentration was added on the surface of the skin. It was incubated at 37˚C for 8 hours. The skin sections were removed from the cell culture flask and rinsed with DPBS and immersion fixed in 4% paraformaldehyde for 4–5 days. Subsequently the tissue was embedded in paraffin and sectioned on a rotary microtome at 7 μm. Tissue sections were collected on glass slides and stained using hematoxylin and eosin, or toluidine blue, or periodic-acid Schiff reagent. Pairs of adjacent sections from each animal not further apart than 100μm were analyzed using an Olympus BX 40 light microscope. Specifically, cross sections of skin samples stained with periodic-acid Schiff reagent were analyzed as these sections featured the morphology of the layers of the epidermis and the underlying basement membrane most clearly.

## 4. Result

### 4.1. Analytical method in HPLC

**Fig 1:**
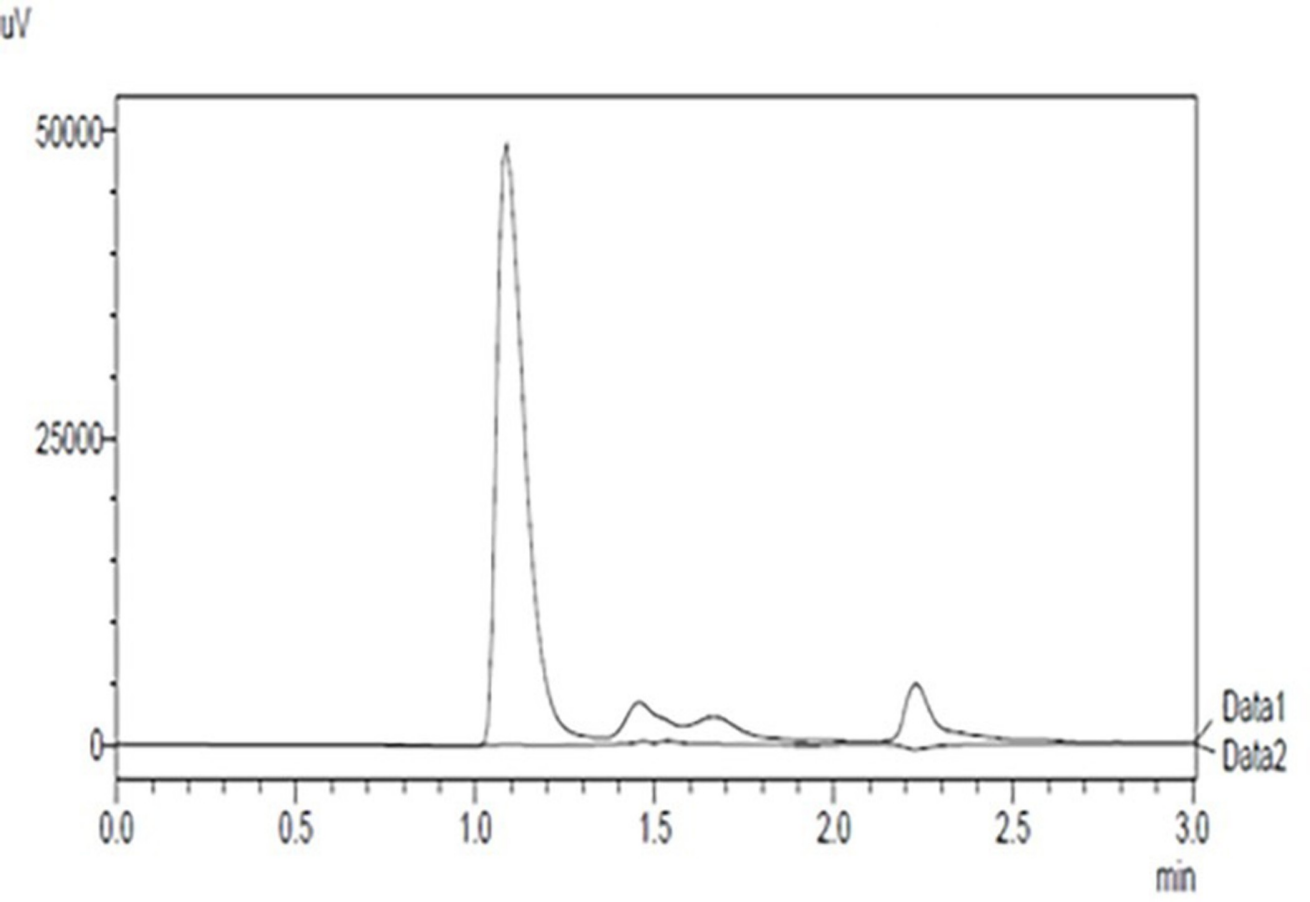
HPLC chromatogram for papain.

The retention time of papain was 1.07 minutes and the run time was 3 minutes. Multiple peaks following the major peak at 1.07 min could be due to impurities present in papain. The method was valid for specificity, linearity, precision, and accuracy. The chromatogram for papain is shown in Fig 1.

### 4.2. Particle size and zeta potential

**Fig 2:**
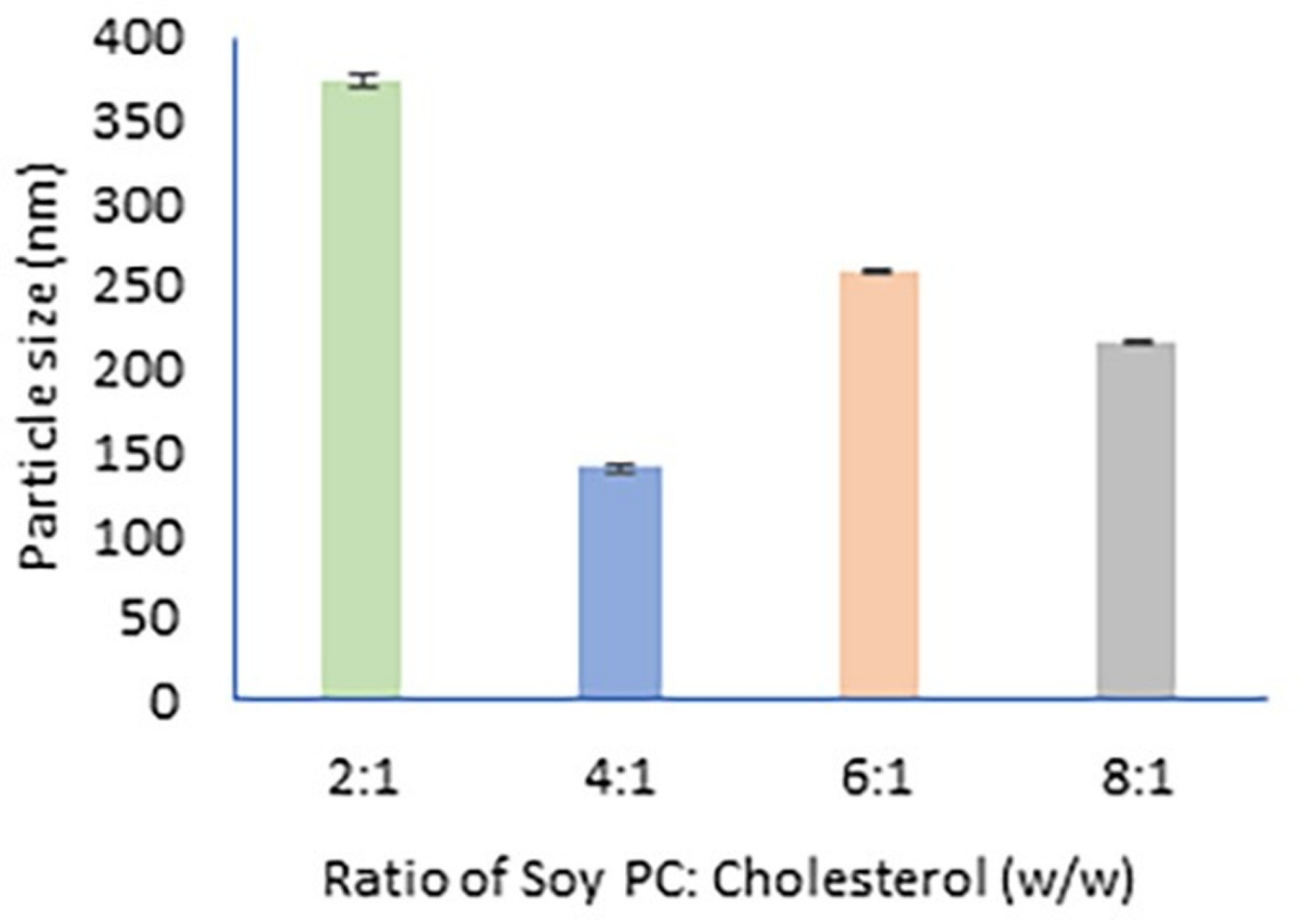
Particle size of liposomes prepared with varying concentrations of soy phosphatidylcholine and cholesterol.

The bilayer of the liposomes is formed by phosphatidylcholine. Cholesterol embeds in the bilayer and increases the rigidity of the bilayer. The addition of cholesterol also affects the size of the particles. Therefore, the optimal ratio of phosphatidylcholine to cholesterol for the particle size of blank liposomes was observed. Analyzing the particle size for different ratios of phosphatidylcholine and cholesterol, we observed that the ideal particle size of 140.2±3.4 nm was given by 4:1 (Soy lecithin: cholesterol). Of the other explored ratios, the particle size was more than 200 nm, which is not ideal for topical delivery. Papain-loaded liposomes were prepared using the 4:1 ratio of phosphatidylcholine and cholesterol. The particle size of papain-loaded liposomes in suspension was found to be 150±2.30 nm with a zeta potential of -50.09±3.66 mV. Similarly, the particle size of papain transferosomes was found to be 158.00±3.50 nm with a zeta potential of -43.51±1.73 mV. A graphical representation of particle size with different concentration of cholesterol is shown in Fig 2.

When papain liposomes and transferosomes were lyophilized, the particle size increased to 475.60±9.60 nm and 238.00±1.10 nm, respectively. This increase in particle size is unacceptable for topical delivery. Therefore, trehalose was used as a cryoprotectant to maintain the particle size even after lyophilization. Of the different concentrations of trehalose explored, 0.04% w/w and 0.5% w/w for transferosomes and liposomes, respectively, were observed. The zeta potential of the particles was similar to that of the particle in suspension.

**Fig 3:**
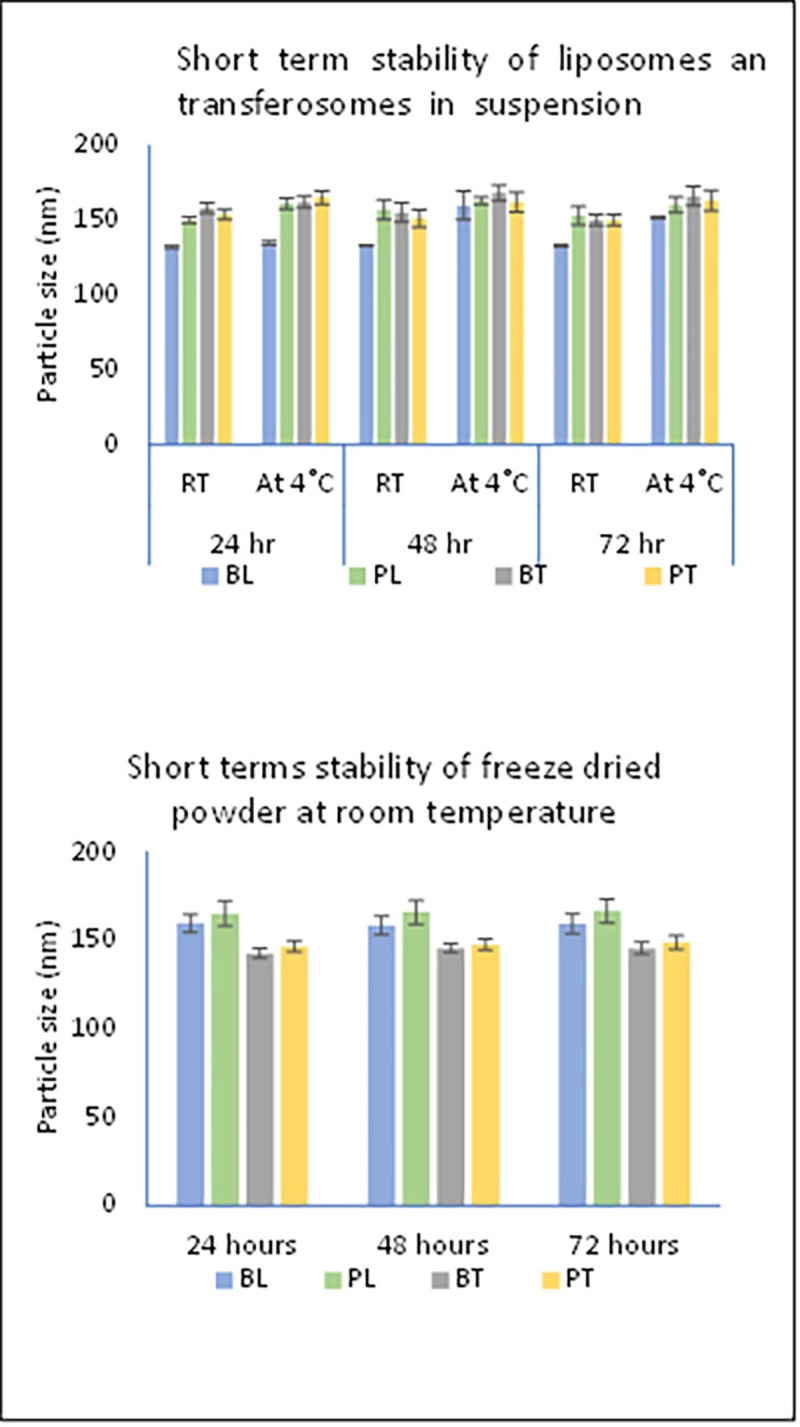
Short term stability of liposomes and transferosomes freeze-dried powder and suspension.

In a short-term physical stability of liposomes and transferosomes, the particle size remained less than 200 nm in the suspension for both blank and papain-loaded vesicles for 72 hours at room temperature and 4°C as shown in Fig 3.

The freeze-dried product was also stable at room temperature for 72 hours. The particle size of liposomes and transferosomes are summarized in Table 1.

Phosphatidyl choline has a positive charge on the choline and negative charge on the phosphate group. In addition, phosphate buffer added in the formulation is an anionic buffer which might have contributed to the net negative charge of these vesicles [33]. Charged liposomes have been reported to have better physical stability compared to the neutral liposomes and the surface charge could reduce the aggregation of particles. A negative charge on the surface of the liposomes has been reported to encourage cell-liposomes interaction with enhanced efficacy [34]. Some studies suggest that cationic liposomes have better chances of penetration through the skin because skin is negatively charged [35, 36]. Some other studies also indicate that since the skin is negatively charged, they show that the intradermal migration of anionic liposomes is more than that of neutral liposomes [37, 38].

### 4.3. Encapsulation efficiency

The encapsulation efficiency of papain-loaded liposomes and transferosomes was determined using a validated method in HPLC. Encapsulation and drug load for papain-loaded liposomes was 96.14±0.295%, and 7.69±0.02%, respectively, and for papain-loaded transferosomes it was 90.98±0.67%, and 7.28±0.05%, respectively. This was determined by subtracting the amount of papain observed in the supernatant after ultracentrifugation from the amount of papain added to the formulation. In the higher order structure of papain, there are hydrophobic regions that are in the vicinity of the globular structure that cause the protein to attract lipids and surfactants [39]. The affinity of papain for hydrophobic entities may have caused a high drug load in the liposomes and transferosomes.

### 4.4. Transmission electron microscopy (TEM) of liposomes and transferosomes

**Fig 4:**
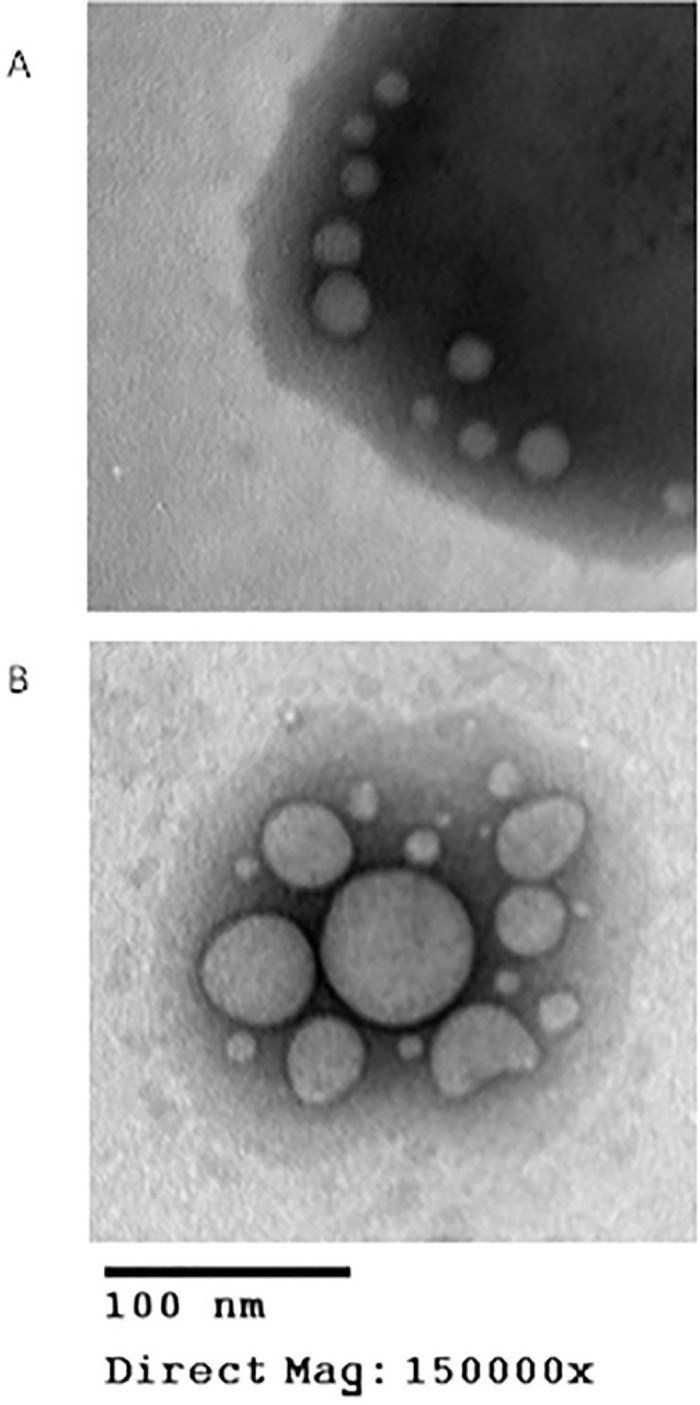
TEM images of (A) papain Transferosomes and (B) papain Liposomes.

Pictures obtained from TEM of liposomes and transferosomes showed that the particles are spherical in shape as shown in Fig 4. Liposomes look like they are collapsing as pictures were taken. This may be due to the exposure to heat from an electron beam during the experiment.

Images from TEM shows smaller particle size as compared to the particle size determined using DLS. TEM measurements require the sample to be in the dry state whereas DLS measures the particle size of a sample in the solvated state. In the solvated state, solvent molecules interact with particles via a variety of non-covalent interactions. While TEM measures the actual particle size, DLS measures the hydrodynamic particle size. Generally, DLS is reported to measure larger particle size due to the presence of dispersant as compared to TEM [40].

### 4.5. Enzymatic activity

**Fig 5:**
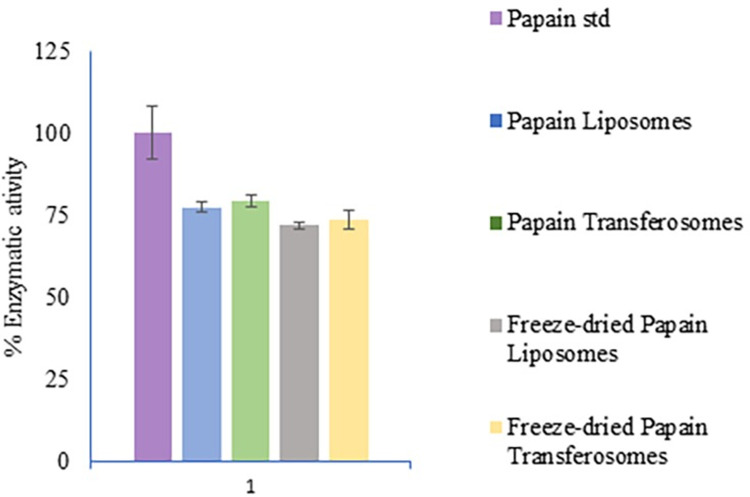
Enzymatic activity of papain loaded liposomes and transferosomes with respect to papain in solution.

The enzyme activity of the liposomes and transferosomes was determined using the protease assay, which quantifies the protein in the formulation. The enzymatic activity of the liposomes in suspension and after freeze drying was 77.55±1.34%, and71.94±1.13%, respectively. The enzymatic activity of the transferosomes in suspension and after freeze drying was 79.15±1.83%, and 73.77±2.99%, respectively. A graphical representation is shown in Fig 5. The percentage is calculated with respect to the enzyme activity of the papain solution. The reduction in enzymatic activity after lyophilization is possibly due to the freezing and drying stresses. The addition of cryoprotectants is known to offer protection from loss of enzymatic activity; however, the low amount of trehalose in the formulation may not have been able to preserve enzymatic activity entirely.

### 4.6. In vitro permeability studies of papain-loaded liposomes and transferosomes on shed snake skin

**Fig 6:**
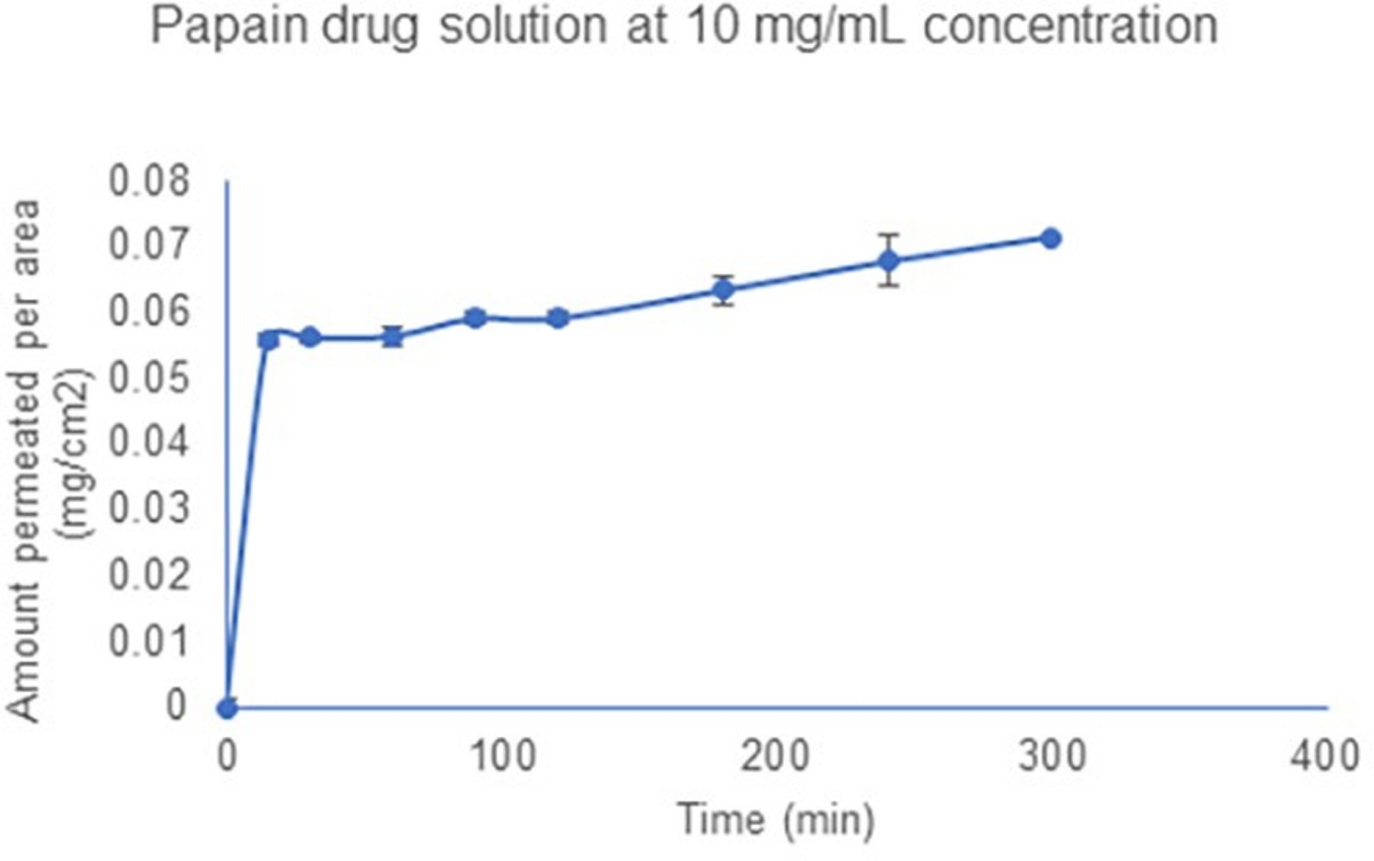
Permeation of papain from aqueous solution.

**Fig 7:**
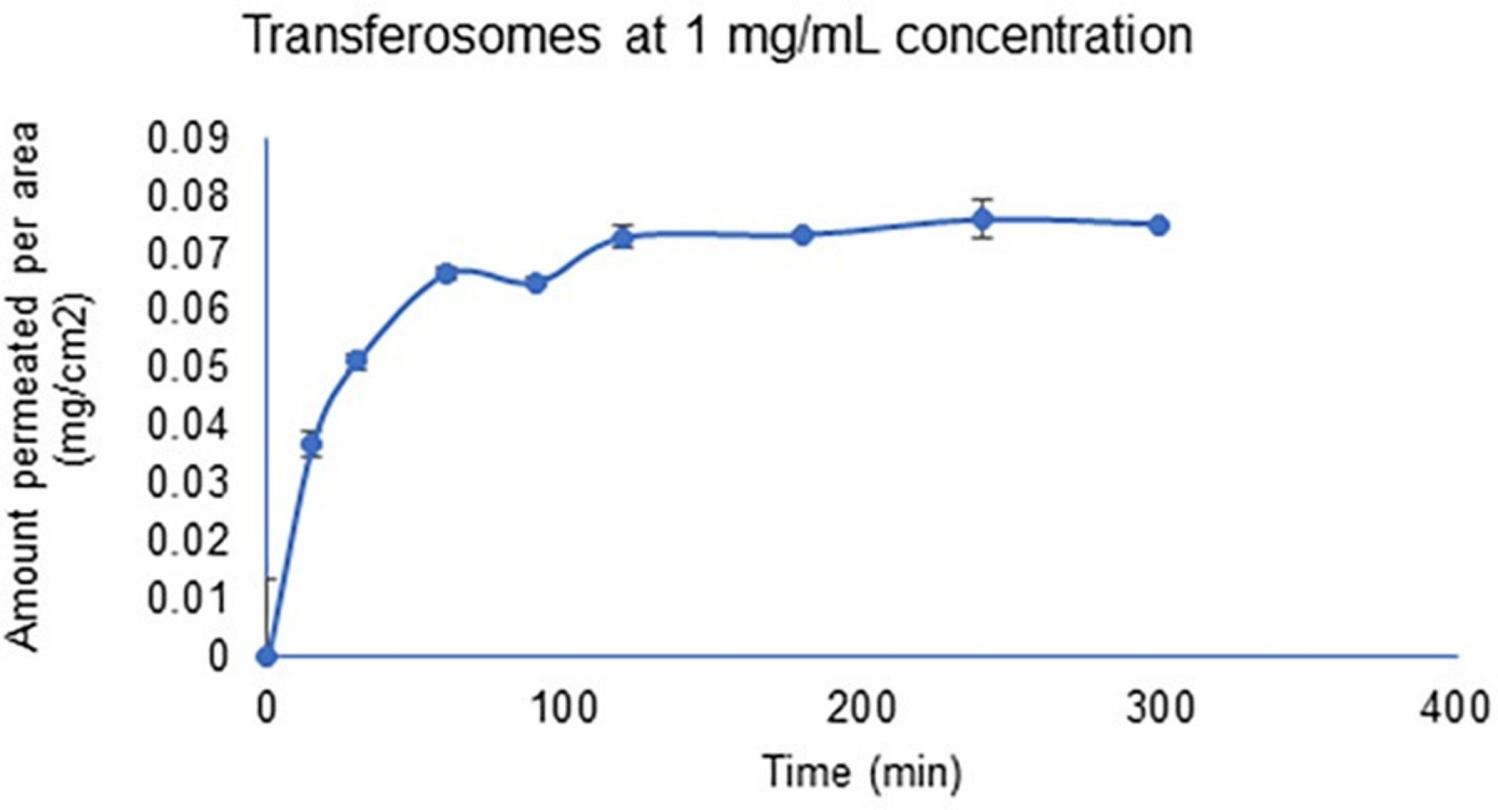
Permeation of papain from papain-loaded transferosomes.

Permeability studies of papain-loaded liposomes and transferosomes were performed on shed-snake skin as a model for the stratum corneum. Papain was not permeated through the shed snake skin by papain-loaded liposomes. In case of papain loaded transferosomes, 68.45±3.4 μg/cm^2^ was permeated through the shed-snake skin over the duration of 5 hours. Papain solution at the same and five times the concentration (5 mg/ml) as papain loaded liposomes and transferosomes did not permeate through the shed-snake skin. However, when the concentration of papain solution in the donor chamber was increased by ten times (to 10 mg/ml), 71.45±3.78 μg/cm^2^ was permeated through the shed-snake skin. The permeation profile for papain and papain-loaded transferosomes is shown in Figs 6 and 7, respectively. The amount of blue dextran on the receiver side was quantified using UV-visible spectroscopy and the membranes permeating blue dextran were considered unfit for the analysis.

Permeability studies were also done on Sprague-Dawley rat skin. It was used as a model for full thickness skin. Papain from solution, papain loaded liposomes and papain loaded transferosomes were not detected in the receiver side by the analytical method in HPLC. The integrity of skin was evaluated by lucifer yellow on the receiver side. Skin sections permeating lucifer yellow were not considered fit for analysis.

### 4.7. MTT cytotoxicity assay

**Fig 8:**
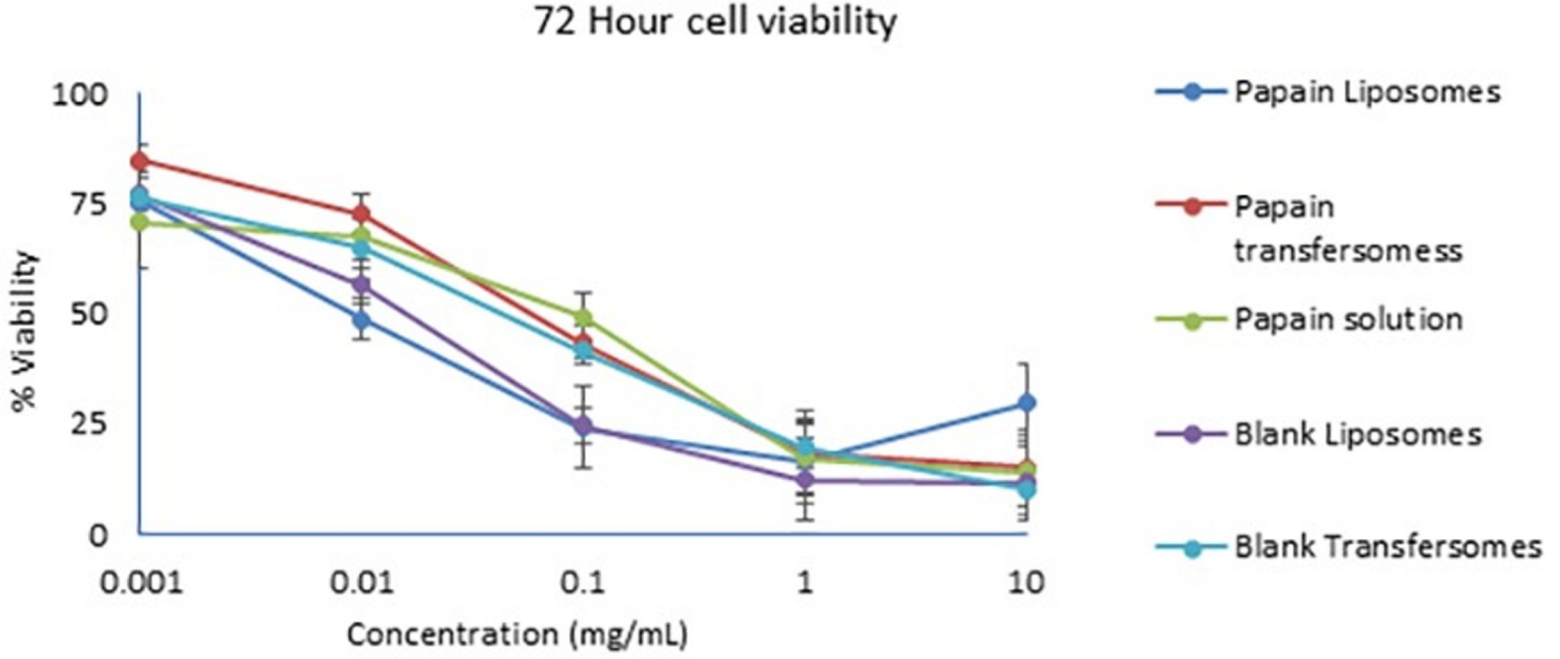
MTT cytotoxicity study of liposomes and transferosomes at 72 hr.

The in vitro cellular toxicity of the drug and formulation was determined on HeLa cells for up to 72 hours. The results showed that more than 50% of the cells were viable at papain concentration of 0.001 mg/ml at 72 hours. Cell viability per concentration for 72 hr is shown in Fig 8. Papain solution, liposomes, and, transferosomes, all showed low cell viability at higher concentrations of papain and nanoparticles. The lipids and the surfactants in the formulation can also cause the toxicity to the cells as they are in very high amounts. The dose of papain is not established. Therefore, the concentrations explored were wide (0.001 mg/ml to 10 mg/ml) and the safe concentrations cannot be extrapolated to draw the therapeutic relevance.

### 4.8. Effects of papain on morphology of epidermis

**Fig 9:**
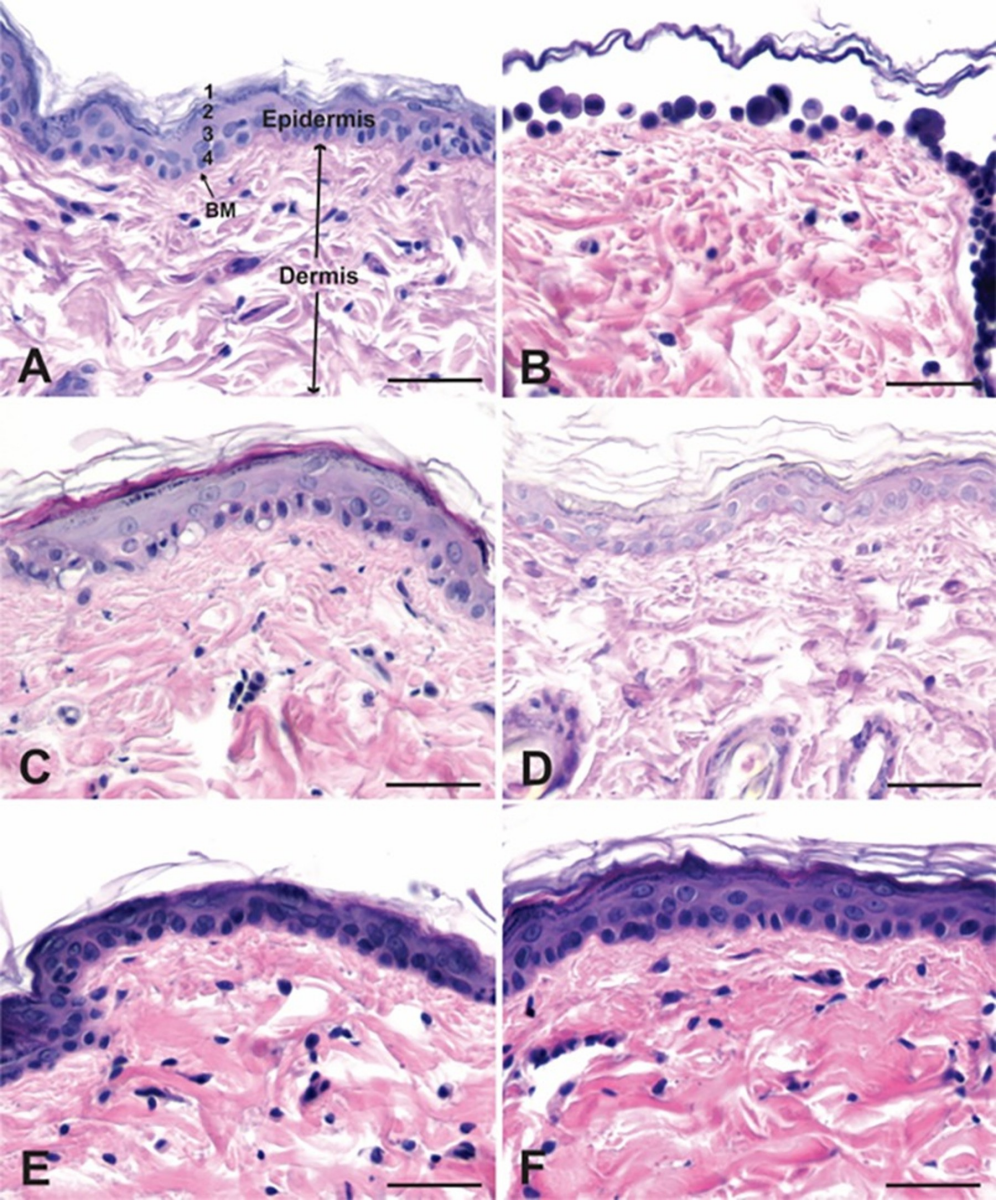
Skin cross sections.

The morphology of the epidermis was examined on 50–60 tissue sections from animals in each group using a light microscope (Table 2). Photographs were taken of tissue sections where the normal layering of the epidermis was disrupted and where the epidermis was missing. The examiner of the tissue sections was blind to the treatment group for each set of slides. The epidermis and underlying basement membrane were absent in patches of skin in tissue sections taken from each of the animals that were exposed to papain in solution, but not from any animals from any of the other treatment groups (Fig 9). In addition to the morphological changes in the epidermis the papain-treated sections were characterized by the presence of large round cells on the surface of the dermis (Fig 9).

**Table 2:** In vivo analysis of skin morphology after papain treatment.

Treatment	Number of animals per group	Average number of tissue sections analyzed per animal (range)
DPBS	3	60 (54–69)
Papain only	3	52 (40–68)
Blank liposomes	3	52 (50–54)
Blank transferosomes	3	50 (42–60)
Papain liposomes	5	53 (46–64)
Papain transferosomes	4	54 (52–56)

## 5. Discussion

Liposomes are known to pass via transepidermal and transappendageal pathways as they permeate the skin. Papain liposomes adhere to the stratum corneum upon application. This encourages the blending of lipids in vesicles with the lipids of the keratinocyte cell membranes in the stratum corneum and can lead to structural changes in the vesicles. The vesicles may not be intact at this point and may start to release their contents. Since papain has both hydrophobic and hydrophilic components in its globular structure, some of the papain may bind with lipids in the stratum corneum. Liposomes may only be localized in the stratum corneum as they lack the flexibility and permeation enhancers to permeate into deeper layers of the epidermis. Further studies are needed to clearly define the mechanism of action of papain liposomes. Transferosomes or the deformable vesicles are purposed to have better permeability by virtue of their stress dependent adaptability which enables them to squeeze between the cells in the stratum corneum despite having similar particle size as liposomes. Cevc and Blume also propose that transferosomes use the transcutaneous hydration gradient to pass intact through the stratum corneum [26, 27]. Transferosomes are functionally different from liposomes due to their biophysical composition. Transferosomes have a relatively high bilayer fluidity which allows them to squeeze between the keratinocytes of the stratum corneum and penetrate through the epidermis to the dermal layer of skin. Liposomes lack this property and are therefore confined to upper layers of the stratum corneum [30, 41, 42].

Liposomes and Transferosomes prepared using the thin-film hydration method generated nanoparticles of desired particle size. Encapsulation efficiency was measured by quantifying papain in supernatant (unencapsulated papain) and subtracting it from the total amount added. The subtraction method of calculating encapsulation efficiency and drug loading provides an estimation of the amount of papain present in the formulation rather than the actual amount. Also, the amount of papain present in the formulation can be either encapsulated or on the outer edge of phospholipids present in nanoparticles. As mentioned above papain has both hydrophilic and hydrophobic zones in its globular structure. Both the core and periphery of liposomes and transferosomes consists of hydrophilic heads of phospholipids which increases the possibility of papain sticking to the outer wall of the nanoparticles. Regardless of the positioning of papain in the nanoparticles, encapsulation efficiency and drug loading provide insight on the amount of papain present in the formulation. The results of enzymatic activity show that around 77% and 79% of protein is present and retains the enzymatic activity in liposomes and transferosomes. The probable cause of the low enzymatic activity as compared to encapsulation efficiency can be due to the conformational changes or orientation of papain in the nanoparticles. During the formation of nanoparticle, the nanoparticle-protein interaction can cause conformational changes that can influence the activity of protein [43]. Following the lysis of nanoparticles, papain may not be oriented in the best way to interact with the substrate, resulting in lower proteolytic activity. Results from the encapsulation efficiency and enzymatic activity studies indicate that papain present in an active form in the formulation and processing parameters does not denature papain.

*In vitro* study of papain-loaded liposomes and transferosomes showed that at a low concentration of 1 mg/ml and 5 mg/ml of papain in the formulation, papain was not delivered across the stratum corneum barrier (shed snake skin model). But at a higher concentration of 10 mg/ml, papain was able to pass through the barrier. According to Fick’s law of diffusion, the amount of material flowing through a cross-sectional area of a barrier in units of time is directly proportional to the concentration gradient. The increase in the concentration gradient contributed to the permeation of papain at higher concentration. Liposomes were also unable to permeate papain through the shed snake skin. This could be due to the limited permeability of liposomes in the outer layer of the stratum corneum. The incorporation of cholesterol adds stiffness to the phospholipid bilayer, making liposomes less likely to permeate through the stratum corneum. However, for transferosomes the addition of tween 80, an edge activator, makes the phospholipid bilayer flexible. For this reason, even at a low concentration of 1 mg/ml, transferosomes were able to deliver a similar amount of papain as the papain solution at high concentration (10 mg/ml). Surfactant such as tween 80 used as edge activators accumulate at the site of stress, destabilize the membrane, making them deformable, and deliver payloads across the stratum corneum. However, in the case of full thickness rat skin, papain in solution, liposomes and transferosomes were not able to deliver papain across it. This could be due to the inverse relation between the amount of material permeated and the distance of movement. Permeation of papain from papain-loaded transferosomes through the shed snake skin (stratum corneum) and not through the full thickness of skin indicates that papain-loaded transferosomes are localized in the epidermal or dermal layer of the skin.

The MTT cytotoxicity assay showed that papain and papain nanoparticles are toxic to HeLa cells. Although, HeLa cells are known to be highly proliferative, the presence of high amounts of lipids and surfactant in the formulation may have influenced the cell behavior and cell organelles like mitochondria [44, 45].

The morphology study of Sprague-Dawley rat skin following the treatment with papain showed the absence of epidermis and the basement membrane. Proteolytic activity of papain caused the proteins that held together the cells in epidermis to breakdown causing the disruption of the epidermal layer. Papain is reported to degrade the tight junction proteins in skin [46]. Blank nanoparticles and papain loaded nanoparticles did not show the same effect as papain on epidermis. Both dermal and epidermal layers were intact in the skin sections treated with blank and papain loaded nanoparticles. This evidence suggests that papain is not safe for administration as it and therefore, needs a medium for the protection of epidermis. Papain liposomes and transferosomes were not able to breakdown the collagen in dermis. From the *in vitro* permeation study, it is evident that papain transferosomes are capable of permeating through the stratum corneum but the amount of papain permeated maybe low to exert any effect. Even though these preliminary toxicity data suggest that these nanovesicles tested are nontoxic, thorough preclinical safety evaluation of the formulations is essential and will be a major part of a future study.

## 6. Conclusion

In conclusion, papain liposomes and transferosomes were formulated and characterized. Papain is capable of tearing down parts of skin and is unsafe for administration as is. Liposomes and transferosomes are useful aids for the transport of papain across epidermis. However, liposomes were not able to permeate through the stratum corneum barrier. Transferosomes, being flexible vesicles, were able to transport papain across the stratum corneum. Low amount of papain permeated or papain being unable to reach the site of action may be the reasons for intact dermis in Sprague-Dawley rat skin.

The future direction for this study can be exploring the effective dose for hypertrophic scars and keloids. Although, this may be challenging as the size, location and severity of scar varies across individuals. Transferosomes and liposomes can be further modified to increase the enzymatic efficiency and permeation across the stratum corneum. A different cell line like fibroblasts can be used to study the cytotoxicity of nanoparticles to understand the toxicity of nanoparticles across different biological models.

## Supporting information

S1 Raw image (TIF)

S2 Raw image (TIF)

S3 Raw image (TIF)

S4 Raw image (TIF)

S1 Graphical abstract (TIF)

## References

[1] Benson HAE, Grice JE, Mohammed Y, Namjoshi S, Roberts MS. Topical and Transdermal Drug Delivery: From Simple Potions to Smart Technologies. Curr Drug Deliv. 2019 Feb 4;16(5):444–60. 30714524 10.2174/1567201816666190201143457 PMC6637104

[2] Michaels AS, Chandrasekaran SK, Shaw JE. Drug permeation through human skin: Theory and invitro experimental measurement. AIChE Journal. 1975;21(5):985–96.

[3] Sahle FF, Gebre-Mariam T, Dobner B, Wohlrab J, Neubert RHH. Skin Diseases Associated with the Depletion of Stratum Corneum Lipids and Stratum Corneum Lipid Substitution Therapy. Skin Pharmacol Physiol [Internet]. 2015 Jan 12 [cited 2020 Sep 25];28(1):42–55. Available from: https://www.karger.com/Article/FullText/360009 doi: 10.1159/000360009 25196193

[4] Marks R. The Stratum Corneum Barrier: The Final Frontier. J Nutr [Internet]. 2004 Aug 1 [cited 2022 Oct 17];134(8):2017S–2021S. Available from: https://academic.oup.com/jn/article/134/8/2017S/4688855 doi: 10.1093/jn/134.8.2017S 15284392

[5] Woodley DT. Distinct Fibroblasts in the Papillary and Reticular Dermis: Implications for Wound Healing [Internet]. Vol. 35, Dermatologic Clinics. Saunders W.B; 2017 [cited 2021 Mar 10]. p. 95–100. Available from: http://www.derm.theclinics.com/article/S0733863516300778/fulltext 27890241 10.1016/j.det.2016.07.004

[6] Bailey AJ, Bazin S, Sims TJ, Le Lous M, Nicoletis C, Delaunay A. Characterization of the collagen of human hypertrophic and normal scars. BBA—Protein Structure. 1975 Oct 20;405(2):412–21. doi: 10.1016/0005-2795(75)90106-3 1180964

[7] Miller MC, Nanchahal J. Advances in the modulation of cutaneous wound healing and scarring [Internet]. Vol. 19, BioDrugs. BioDrugs; 2005 [cited 2021 Mar 1]. p. 363–81. Available from: https://pubmed.ncbi.nlm.nih.gov/16392889/ doi: 10.2165/00063030-200519060-00004 16392889

[8] Zhu Z, Ding J, Tredget EE. The molecular basis of hypertrophic scars. Burns Trauma [Internet]. 2016 Dec 1 [cited 2021 Mar 1];4. Available from: https://pubmed.ncbi.nlm.nih.gov/27574672/27574672 10.1186/s41038-015-0026-4 PMC4963951

[9] Alster TS, Tanzi EL. Hypertrophic scars and keloids: Etiology and management [Internet]. Vol. 4, American Journal of Clinical Dermatology. Springer; 2003 [cited 2020 Nov 19]. p. 235–43. Available from: https://link.springer.com/article/10.2165/00128071-200304040-00003 12680802 10.2165/00128071-200304040-00003

[10] Family A, Juckett G, Hartman-Adams H. Management of Keloids and Hypertrophic Scars [Internet]. Vol. 80, American Family Physician. 2009 Aug [cited 2021 May 6]. Available from: www.aafp.org/afp 19621835

[11] Carswell L, Borger J. Hypertrophic Scarring Keloids [Internet]. StatPearls. StatPearls Publishing; 2019 [cited 2021 Mar 2]. Available from: http://www.ncbi.nlm.nih.gov/pubmed/30725743

[12] Darzi MA, Chowdri NA, Kaul SK, Khan M. Evaluation of various methods of treating keloids and hypertrophic scars: a 10-year follow-up study. Br J Plast Surg. 1992 Jan 1;45(5):374–9. doi: 10.1016/0007-1226(92)90008-l 1638291

[13] Lawrence WT. In Search of the Optimal Treatment of Keloids: Report of a Series and a Review of the Literature. Ann Plast Surg [Internet]. 1991 Aug 1 [cited 2021 Mar 4];27(2):164–78. Available from: http://journals.lww.com/00000637-199108000-00012 doi: 10.1097/00000637-199108000-00012 1835334

[14] Ogawa R. Keloid and Hypertrophic Scars Are the Result of Chronic Inflammation in the Reticular Dermis. Int J Mol Sci [Internet]. 2017 Mar 10 [cited 2020 Dec 5];18(3):606. Available from: http://www.mdpi.com/1422-0067/18/3/606 28287424 10.3390/ijms18030606 PMC5372622

[15] Mitchel REJ, Chaiken IM, Smith EL. The Complete Amino Acid Sequence of Papain. Journal of Biological Chemistry. 1970 Jul 25;245(14):3485–92. 5470818

[16] Budama-Kilinc Y, Cakir-Koc R, Kecel-Gunduz S, Zorlu T, Kokcu Y, Bicak B, et al. Papain Loaded Poly(ε-Caprolactone) Nanoparticles: In-silico and In-Vitro Studies. J Fluoresc. 2018 Sep 1;28(5):1127–42. 30097974 10.1007/s10895-018-2276-6

[17] Müller A, Barat S, Chen X, Bui KC, Bozko P, Malek NP, et al. Comparative study of antitumor effects of bromelain and papain in human cholangiocarcinoma cell lines. Int J Oncol [Internet]. 2016 May 1 [cited 2021 Mar 7];48(5):2025–34. Available from: http://www.spandidos-publications.com/10.3892/ijo.2016.3411/abstract doi: 10.3892/ijo.2016.3411 26935541

[18] Abdel-Hamid M, Goda HA, De Gobba C, Jenssen H, Osman A. Antibacterial activity of papain hydrolysed camel whey and its fractions. Int Dairy J. 2016 Oct 1;61:91–8.

[19] Papain | Sigma-Aldrich [Internet]. [cited 2021 May 23]. Available from: https://www.sigmaaldrich.com/life-science/metabolomics/enzyme-explorer/analytical-enzymes/papain.html

[20] Lowe G, Yuthavong Y. Kinetic specificity in papain-catalysed hydrolyses. Biochem J [Internet]. 1971 Aug 1 [cited 2021 May 26];124(1):107–15. Available from: /biochemj/article/124/1/107/17257/Kinetic-specificity-in-papain-catalysed-hydrolyses doi: 10.1042/bj1240107 5126466 PMC1177119

[21] Sapkota R, Dash A. Liposomes and Transfersomes for the Topical Delivery of Papain in the Treatment of Hypertrophic Scars [Internet]. 2021 [cited 2022 Oct 19]. Available from: http://dspace.creighton.edu:8080/xmlui/handle/10504/130604

[22] Bos JD, Meinardi MMHM. The 500 Dalton rule for the skin penetration of chemical compounds and drugs. Exp Dermatol [Internet]. 2000 Jun [cited 2023 Jan 15];9(3):165–9. Available from: https://pubmed.ncbi.nlm.nih.gov/10839713/ doi: 10.1034/j.1600-0625.2000.009003165.x 10839713

[23] Sessa G, Weissmann G. Phospholipid spherules (liposomes) as a model for biological membranes. Vol. 9, Journal Lipid Research. 1968. 5646182

[24] Gregoriadis G, Ryman BE. Liposomes as carriers of enzymes or drugs: a new approach to the treatment of storage diseases. Biochemical Journal [Internet]. 1971 Oct 1 [cited 2021 Mar 10];124(5):58P-58P. Available from: /biochemj/article/124/5/58P/8318/Liposomes-as-carriers-of-enzymes-or-drugs-a-new doi: 10.1042/bj1240058p 5130994 PMC1177319

[25] Gregoriadis G. Drug entrapment in liposomes. FEBS Lett [Internet]. 1973 Nov 1 [cited 2021 Mar 10];36(3):292–6. Available from: http://doi.wiley.com/10.1016/0014-5793(73)80394-1 doi: 10.1016/0014-5793(73)80394-1 4763309

[26] Transfersomes Cevc G., liposomes and other lipid suspensions on the skin: Permeation enhancement, vesicle penetration, and transdermal drug delivery. Crit Rev Ther Drug Carrier Syst [Internet]. 1996 [cited 2021 Mar 10];13(3–4):257–388. Available from: http://www.dl.begellhouse.com/journals/3667c4ae6e8fd136,401f5e031bfcf4f2,372a6d69178d22ce.html 9016383 10.1615/critrevtherdrugcarriersyst.v13.i3-4.30

[27] Cevc G, Blume G. Lipid vesicles penetrate into intact skin owing to the transdermal osmotic gradients and hydration force. Vol. 1104, Biochimica et Biophysica Acta. 1992;111:226–32. 10.1016/0005-2736(92)90154-e 1550849

[28] Cevc G, Blume G. New, highly efficient formulation of diclofenac for the topical, transdermal administration in ultradeformable drug carriers, Transfersomes. Biochim Biophys Acta Biomembr. 2001 Oct 1;1514(2):191–205. doi: 10.1016/s0005-2736(01)00369-8 11557020

[29] Planas ME, Gonzalez P, Rodriguez L, Sanchez S, Cevc G. Noninvasive Percutaneous Induction of Topical Analgesia by a New Type of Drug Carrier, and Prolongation of Local Pain Insensitivity by Anesthetic Liposomes. Anesth Analg [Internet]. 1992 Oct 1 [cited 2020 Aug 16];75(4):615???621. Available from: http://journals.lww.com/00000539-199210000-00027 doi: 10.1213/00000539-199210000-00027 1530176

[30] Sapkota R, Dash AK. Liposomes and transferosomes: a breakthrough in topical and transdermal delivery. https://doi.org/104155/tde-2020-0122 [Internet]. 2021 Feb 15 [cited 2021 Oct 11];12(2):145–58. Available from: https://www.future-science.com/doi/abs/10.4155/tde-2020-0122 10.4155/tde-2020-0122 33583219

[31] Ahad A, Al-Saleh AA, Al-Mohizea AM, Al-Jenoobi FI, Raish M, Yassin AEB, et al. Formulation and characterization of novel soft nanovesicles for enhanced transdermal delivery of eprosartan mesylate. Saudi Pharmaceutical Journal. 2017 Nov 1;25(7):1040–6. doi: 10.1016/j.jsps.2017.01.006 29158713 PMC5681305

[32] El Zaafarany GM, Awad GAS, Holayel SM, Mortada ND. Role of edge activators and surface charge in developing ultradeformable vesicles with enhanced skin delivery. Int J Pharm [Internet]. 2010 [cited 2021 Apr 14];397(1–2):164–72. Available from: https://reader.elsevier.com/reader/sd/pii/S037851731000462X?token=281B293DAEF71398BCADE09290022A43F9FD742C470F0EE5C9BE005A64CD0B70CCE9EC36F019781C2F550F2DA1D92CC7&originRegion=us-east-1&originCreation=20210415163323 doi: 10.1016/j.ijpharm.2010.06.034 20599487

[33] Gayán E, Condón S, Álvarez I, Nabakabaya M, Mackey B. Effect of pressure-induced changes in the ionization equilibria of buffers on inactivation of Escherichia coli and staphylococcus aureus by high hydrostatic pressure. Appl Environ Microbiol. 2013;79(13). doi: 10.1128/AEM.00469-13 23624471 PMC3697583

[34] Sharma A, Sharma US. Liposomes in drug delivery: Progress and limitations. Int J Pharm. 1997 Aug 26;154(2):123–40.

[35] Ibaraki H, Kanazawa T. In Vivo Topical and Systemic Distribution Kinetics of Liposomes with Various Properties for Application to Drug Delivery Systems. Sensors and Materials [Internet]. 2022 [cited 2022 Sep 20];34(3):987–1005. Available from: 10.18494/SAM3673

[36] Kitagawa S, Bulletin MKC and P, 2006 undefined. Enhanced delivery of retinoic acid to skin by cationic liposomes. jstage.jst.go.jp [Internet]. 2006 [cited 2022 Sep 19]; Available from: https://www.jstage.jst.go.jp/article/cpb/54/2/54_2_242/_article/-char/ja/ doi: 10.1248/cpb.54.242 16462074

[37] Sinico C, Manconi M, Peppi M, Lai F, Valenti D, Fadda AM. Liposomes as carriers for dermal delivery of tretinoin: In vitro evaluation of drug permeation and vesicle-skin interaction. Journal of Controlled Release. 2005 Mar 2;103(1):123–36. doi: 10.1016/j.jconrel.2004.11.020 15710506

[38] Gillet A, Lecomte F, Hubert P, Ducat E, Evrard B, Piel G. Skin penetration behaviour of liposomes as a function of their composition. European Journal of Pharmaceutics and Biopharmaceutics. 2011 Sep 1;79(1):43–53. doi: 10.1016/j.ejpb.2011.01.011 21272638

[39] Amri E, Mamboya F. PAPAIN, A PLANT ENZYME OF BIOLOGICAL IMPORTANCE: A REVIEW. Am J Biochem Biotechnol [Internet]. 2012 [cited 2021 Apr 13];8(2):99–104. Available from: http://www.thescipub.com/ajbb.toc

[40] Souza T. G. F. et a. A comparison of TEM and DLS methods to characterize size distribution of ceramic nanoparticles. J Phys: Conf. 2016.

[41] Kim AR, An HJ, Jang ES, Lee JD, Park SN. Preparation, Physical Characterization, and In Vitro Skin Permeation of Deformable Liposomes Loaded with Taxifolin and Taxifolin Tetraoctanoate. European Journal of Lipid Science and Technology [Internet]. 2019 Jun 7 [cited 2020 May 31];121(6):1800501. Available from: https://onlinelibrary.wiley.com/doi/abs/10.1002/ejlt.201800501

[42] Tiwari G, Tiwari R, Singh R, Rai AK. Ultra-deformable Liposomes as Flexible Nanovesicular Carrier to Penetrate Versatile Drugs Transdermally. Nanoscience & Nanotechnology-Asia. 2018 Aug 23; 10(1):12–20.

[43] Saptarshi SR, Duschl A, Lopata AL. Interaction of nanoparticles with proteins: relation to bio-reactivity of the nanoparticle. J Nanobiotechnology [Internet]. 2013 Jul 19 [cited 2023 Jan 15];11(1):26. Available from: /pmc/articles/PMC3720198/ doi: 10.1186/1477-3155-11-26 23870291 PMC3720198

[44] Koley D, Bard AJ. Triton X-100 concentration effects on membrane permeability of a single HeLa cell by scanning electrochemical microscopy (SECM). Proc Natl Acad Sci U S A [Internet]. 2010 Sep 28 [cited 2023 Jan 15];107(39):16783–7. Available from: /pmc/articles/PMC2947864/ doi: 10.1073/pnas.1011614107 20837548 PMC2947864

[45] Falchi AM, Rosa A, Atzeri A, Incani A, Lampis S, Meli V, et al. Effects of monoolein-based cubosome formulations on lipid droplets and mitochondria of HeLa cells. Cite this: Toxicol Res [Internet]. 2015 [cited 2023 Jan 15];4:1025. Available from: www.rsc.org/toxicology

[46] Stremnitzer C, Manzano-Szalai K, Willensdorfer A, Starkl P, Pieper M, König P, et al. Papain degrades tight junction proteins of human keratinocytes in vitro and sensitizes C57BL/6 mice via the skin independent of its enzymatic activity or TLR4 activation. Journal of Investigative Dermatology. 2015 Jul 18;135(7):1790–800. doi: 10.1038/jid.2015.58 25705851 PMC4471117

