# Supplementary figures and images for: Liposomes and transferosomes in the delivery of papain for the treatment of keloids and hypertrophic scars
Source: PLoS One. 2023 Dec 15;18(12):e0290224. doi: 10.1371/journal.pone.0290224 (PMC10723692; doi:10.1371/journal.pone.0290224)

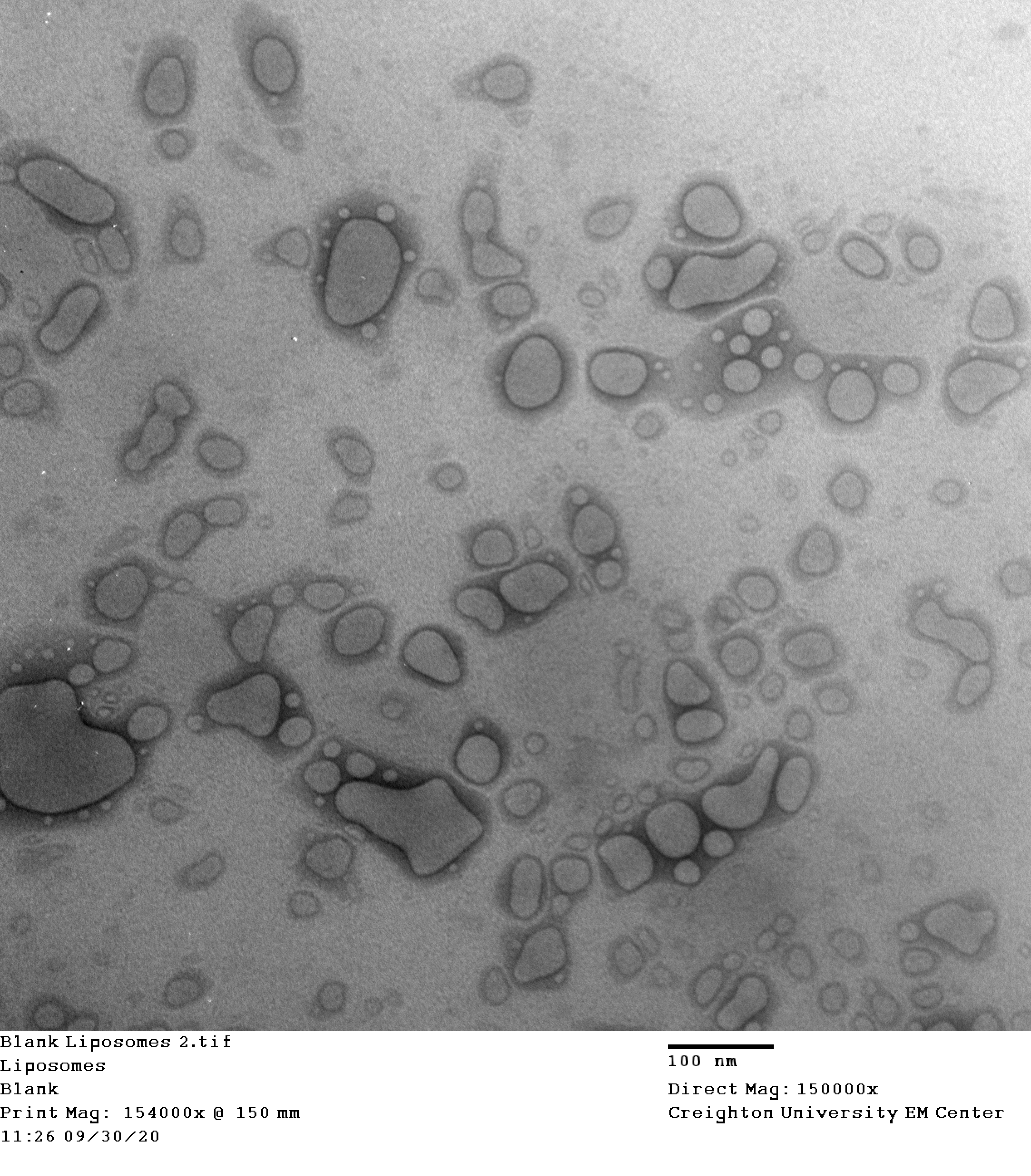

Supplement: S1 Raw image — (TIF) [file pone.0290224.s001.tif]

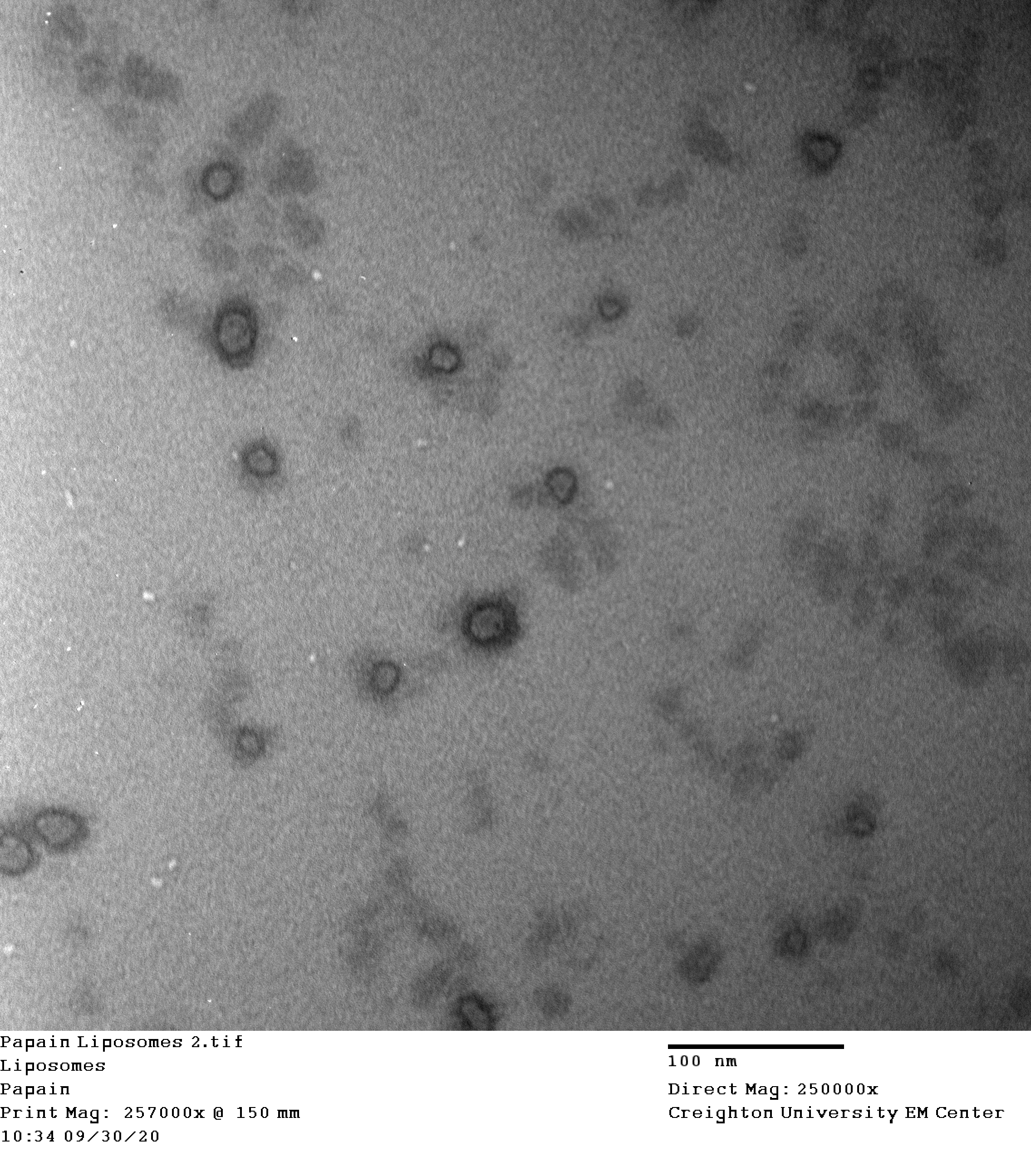

Supplement: S2 Raw image — (TIF) [file pone.0290224.s002.tif]

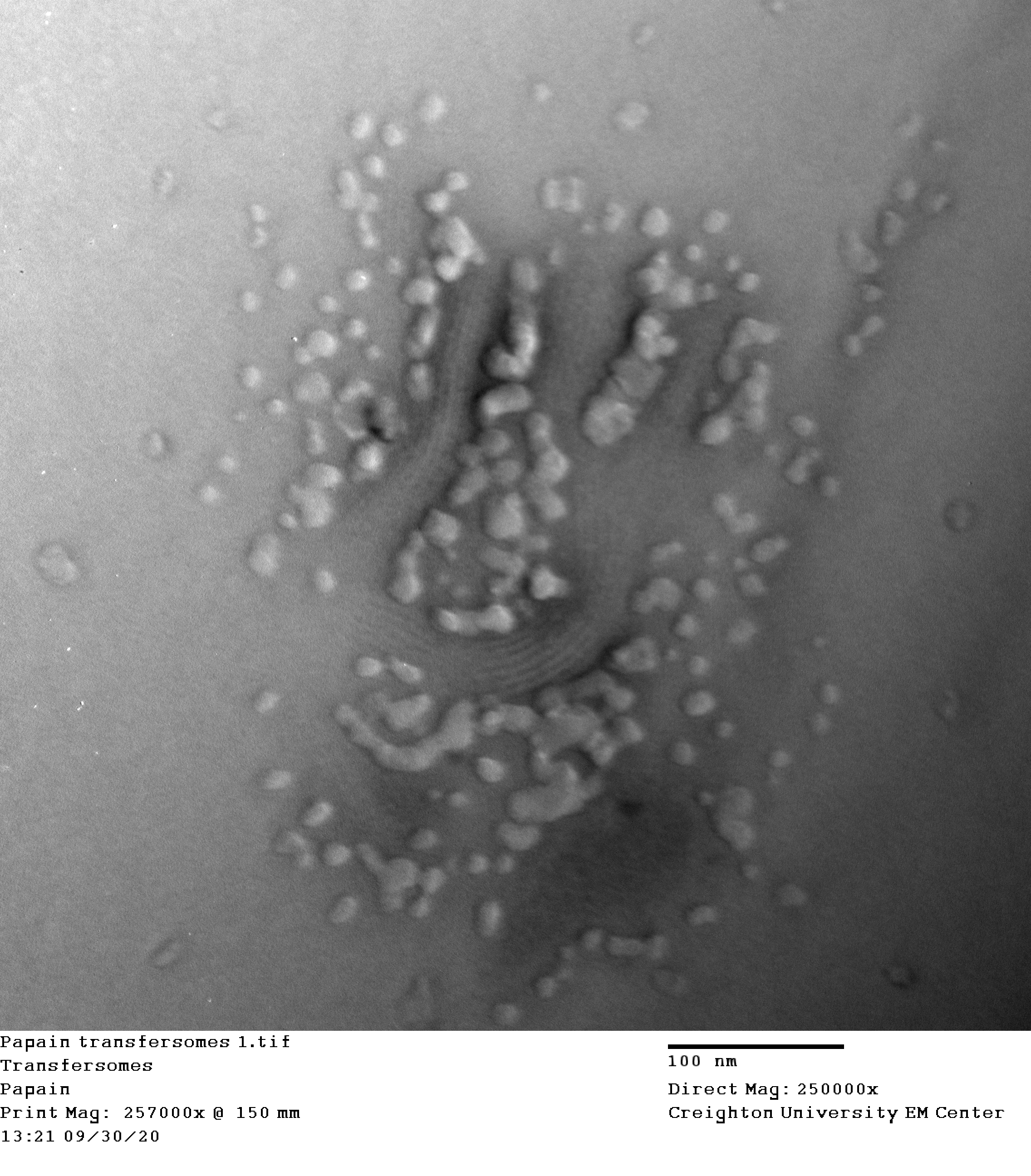

Supplement: S3 Raw image — (TIF) [file pone.0290224.s003.tif]

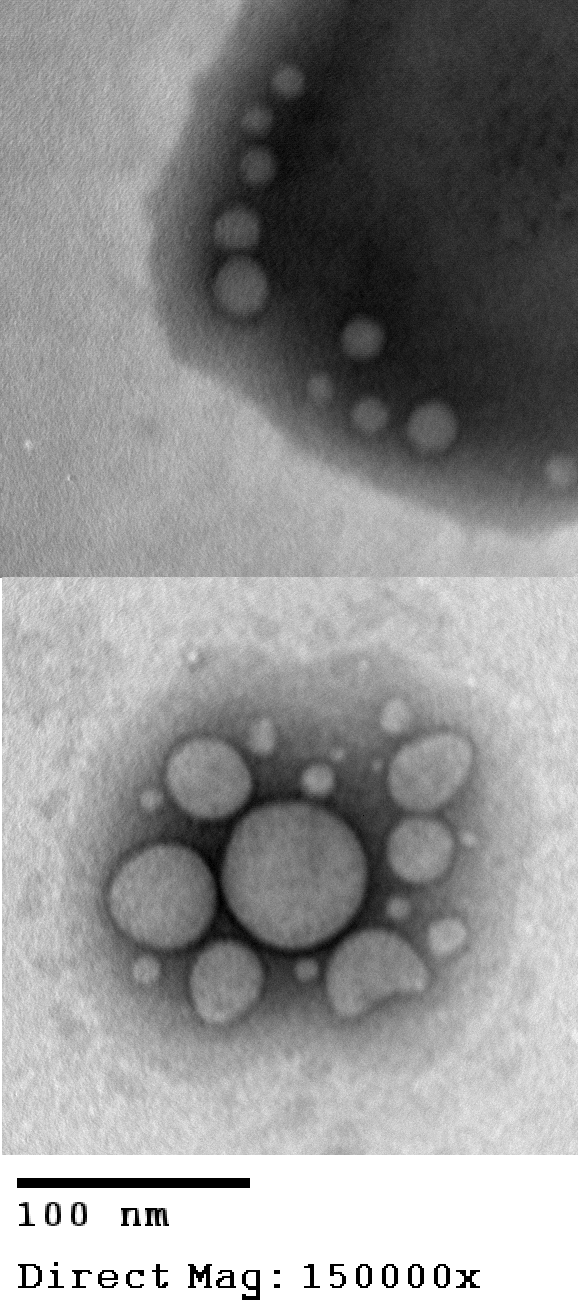

Supplement: S4 Raw image — (TIF) [file pone.0290224.s004.tif]

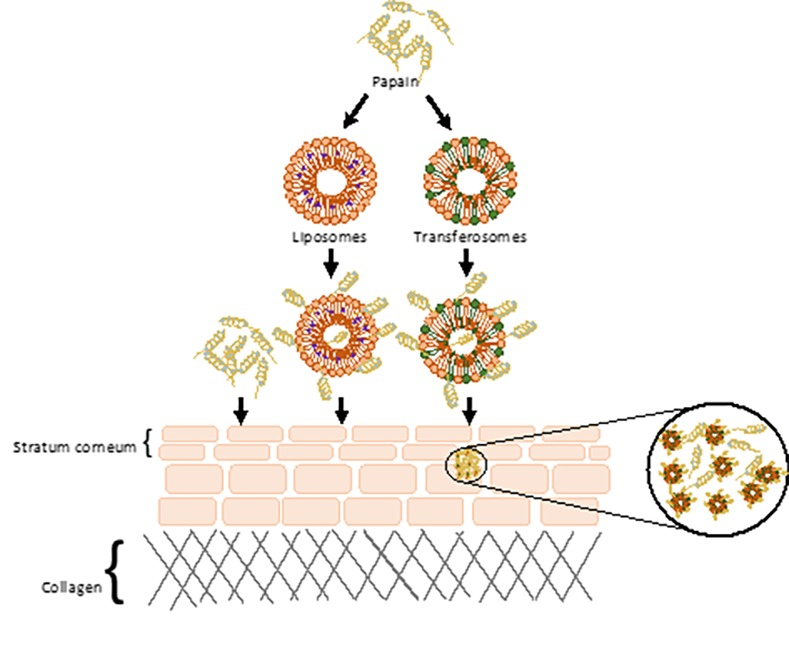

Supplement: S1 Graphical abstract — (TIF) [file pone.0290224.s005.tif]
